# The Complement Binding and Inhibitory Protein CbiA of *Borrelia miyamotoi* Degrades Extracellular Matrix Components by Interacting with Plasmin(ogen)

**DOI:** 10.3389/fcimb.2018.00023

**Published:** 2018-02-02

**Authors:** Ngoc T. T. Nguyen, Florian Röttgerding, Gayatri Devraj, Yi-Pin Lin, Arno Koenigs, Peter Kraiczy

**Affiliations:** ^1^Institute of Medical Microbiology and Infection Control, University Hospital of Frankfurt, Frankfurt, Germany; ^2^Division of Infectious Diseases, New York State Department of Health, Wadsworth Center, Albany, NY, United States; ^3^VIROTECH Diagnostics GmbH, Rüsselsheim, Germany

**Keywords:** lyme disease, spirochetes, borrelia, *Borrelia miyamotoi*, plasminogen, fibrinolysis

## Abstract

The emerging relapsing fever spirochete *Borrelia* (*B*.) *miyamotoi* is transmitted by ixodid ticks and causes the so-called hard tick-borne relapsing fever or *B. miyamotoi* disease (BMD). More recently, we identified a surface-exposed molecule, CbiA exhibiting complement binding and inhibitory capacity and rendering spirochetes resistant to complement-mediated lysis. To gain deeper insight into the molecular principles of *B. miyamotoi*-host interaction, we examined CbiA as a plasmin(ogen) receptor that enables *B. miyamotoi* to interact with the serine protease plasmin(ogen). Recombinant CbiA was able to bind plasminogen in a dose-dependent fashion. Moreover, lysine residues appear to play a crucial role in the protein-protein interaction as binding of plasminogen was inhibited by the lysine analog tranexamic acid as well as increasing ionic strength. Of relevance, plasminogen bound to CbiA can be converted by urokinase-type plasminogen activator (uPa) to active plasmin which cleaved both, the chromogenic substrate S-2251 and its physiologic substrate fibrinogen. Concerning the involvement of specific amino acids in the interaction with plasminogen, lysine residues located at the C-terminus are frequently involved in the binding as reported for various other plasminogen-interacting proteins of Lyme disease spirochetes. Lysine residues located within the C-terminal domain were substituted with alanine to generate single, double, triple, and quadruple point mutants. However, binding of plasminogen to the mutated CbiA proteins was not affected, suggesting that lysine residues distant from the C-terminus might be involved in the interaction.

## Introduction

*Borrelia (B.) miyamotoi*, an emerging relapsing fever spirochete, is transmitted by hard-bodied, ixodid ticks and causes systemic infections accompanied with symptoms similar to relapsing fever, including headache, fatigue, chills, myalgia, arthralgia, nausea, and high-grade fever with possible relapses (Platonov et al., 2011; Molloy et al., 2015; Jobe et al., 2016; Stone and Brissette, 2017) leading to the description of a new entity termed hard tick-borne relapsing fever (HTBRF) or *B. miyamotoi* disease (BMD) (Krause and Barbour, 2015; Telford et al., 2015). First cases of patients with nonspecific febrile symptoms were described in 2011 in central Russia (Platonov et al., 2011), 15 years after the first discovery of *B. miyamotoi* in *Ixodes persulcatus* in Hokkaido, Japan (Fukunaga et al., 1995). Although rare, more severe clinical cases of chronic courses with involvement of the central nervous system have been reported in immunocompromised patients both in the US and in the Netherlands (Gugliotta et al., 2013; Hovius et al., 2013; Boden et al., 2016).

*Borrelia miyamotoi* originated from different geographical regions, e.g., Asia, Siberia, Europe, and North America exhibit considerable genetic diversity, though by contrast extremely low genetic variability is observed among strains isolated from the same region, allowing discrimination of at least three separate clades and classification of the *B. miyamotoi* sensu lato complex (Bunikis et al., 2004; Barbour, 2014; Takano et al., 2014; Mukhacheva et al., 2015). As expected, *B. miyamotoi* occurs sympatrically with spirochetes belonging to the *Borrelia burgdorferi* sensu lato complex in Asia (Fukunaga et al., 1995), North America (Scoles et al., 2001; Barbour et al., 2009), and Europe (Richter et al., 2003) and therefore can be potentially co-transmitted during the blood meal by the same tick that carries multiple vector-borne pathogens, e.g., *Borrelia* spp., *Anaplasma phagocytophilum*, and *Babesia microti*.

The ability of pathogenic microorganisms to overcome innate immunity and to disseminate in the human host is a prerequisite for the establishment of an infection, often accompanied with the progression of the disease by dissemination of the invaders via the circulatory system. In order to establish an infection, the spirochetes must overcome the host complement system as an essential part of innate immunity and indeed *B. miyamotoi* exhibit a remarkable resistance to complement-mediated killing (Teegler et al., 2014; Wagemakers et al., 2014; Margos et al., 2015). More recently, CbiA, a complement-inhibitory protein, has been identified that interacts with complement in multiple ways, binding distinct complement components including key complement regulator Factor H (FH), C3, C3b, C4b, and C5 and thereby terminating activation of distinct complement pathways (Röttgerding et al., 2017).

Spirochetal surface-exposed molecules often exhibit multiple biological functions to directly or indirectly regulate or inhibit host defense systems or in terms of the fibrinolytic system, recruit plasminogen for their own benefit to facilitate dissemination and migration into extravascular tissues. The latter is accomplished by utilizing the proteolytic activity of plasmin, thereby enhancing spirochetemia in the host (Coleman et al., 1997; Gebbia et al., 1999; Vieira and Nascimento, 2016). Concerning relapsing fever and Lyme disease spirochetes, a number of outer surface proteins including BhCRASP-1, HcpA, BpcA, CspA, CspZ, ErpA, ErpP, ErpC, Erp63, and OspC have been characterized that display dual binding properties to plasminogen and complement (Rossmann et al., 2007; Brissette et al., 2009; Grosskinsky et al., 2009; Hallström et al., 2010; Schott et al., 2010; Seling et al., 2010; Hammerschmidt et al., 2014; Caine et al., 2017).

Plasminogen, a 92-kDa glycoprotein, is synthesized in the liver and present in human serum and many extravascular fluids. The inactive proenzyme consists of an N-terminal pre-activation peptide, five lysine-binding, disulfide-bonded kringle domains (K1–K5) and a C-terminal serine protease domain (Ponting et al., 1992). Plasminogen is converted to active plasmin through proteolytic cleavage by tissue-type plasminogen activator (tPA) or urokinase-type plasminogen activator (uPA) (Dano et al., 1985). Plasmin exhibits a relatively low substrate specificity and in addition to its physiological substrate fibrinogen, is able to degrade constituents of the extracellular matrix such as fibronectin, vitronectin, laminin, heparan sulfate proteoglycans and inactive precursors of various matrix metalloproteases (Barthel et al., 2012a).

Neurological manifestations caused by relapsing fever spirochetes invading the CNS have frequently been reported since 1918 where Leboeuf and Gambier described two cases of CSF infection by spirochetes in Brazzaville, sub-Saharan Africa (Republic Congo) (Leboeuf and Gambier, 1918). Besides encephalitis, myelitis, and radiculitis, meningismus and facial palsy are the most frequently described clinical complications predominantly caused by *B. duttonii, B. turicatae*, and *B. recurrentis*, and to some extent also by *B. hermsii, B. hispanica*, and *B. persica* (Cadavid and Barbour, 1998). In addition to the tick-borne relapsing fever (TBRF) or louse-borne relapsing fever (LBRF) spirochetes, *B. miyamotoi* has been detected as the etiological agent of meningoencephalitis in immunocompromised patients (Gugliotta et al., 2013; Hovius et al., 2013; Boden et al., 2016), suggesting dissemination of spirochetes through the cardiovascular system and penetration of the blood-brain-barrier to reach the central nervous system. How *B. miyamotoi* interacts with human endothelial cells of the vasculature, in particular with neuronal cells, and which underlying mechanisms are involved in supporting these processes has not been analyzed so far.

To gain deeper insight into the molecular principles of *B. miyamotoi*-host interaction, we investigated CbiA as a novel plasmin(ogen) receptor that enables *B. miyamotoi* to degrade extracellular matrix components for transmigration and penetration to deeper tissues.

## Materials and methods

### Bacterial strains and culture conditions

*Escherichia (E.) coli* JM109 cells (Promega, Mannheim, Germany) used for production of Hexahistidine (His_6_)-tagged proteins were grown in yeast tryptone broth at 37°C.

### Proteins and antisera

Human glu-plasminogen was obtained from Haematologic Technologies (Essex Junction, VT, USA). Plasminogen was activated to plasmin using urokinase plasminogen activator (uPA) from Merck Millipore, Darmstadt, Germany. Both the chromogenic substrate S-2251 (D-Val-Leu-Lys *p*-nitroanilide dihydrochloride) and fibrinogen were purchased from Sigma-Aldrich (Steinheim, Germany). Goat anti-fibrinogen antiserum was purchased from Acris Antibodies (Herford, Germany), mouse anti-His antiserum was obtained from GE Healthcare (Munich, Germany). Horseradish peroxidase (HRP)-conjugated immunoglobulins were purchased from Dako (Hamburg, Germany).

### Recombinant proteins and generation of CbiA mutants by site-directed mutagenesis

His_6_-tagged CbiA originated from *B. miyamotoi* and BBA70, DbpA, and CspA from *B. burgdorferi* were produced as previously described (Benoit et al., 2011; Koenigs et al., 2013; Hammerschmidt et al., 2014; Röttgerding et al., 2017). To generate His_6_-tagged CbiA proteins with single, double, triple, and quadruple amino acid substitutions, PCR was performed with primers designed for site-directed mutagenesis (Supplementary Table 1; Kraiczy et al., 2009). Plasmid pQE-CbiA (Röttgerding et al., 2017) was used as template for introducing single amino acid substitutions R145A, K153A, K154A, R156A, K162A, R185A, and K188A. For double substitutions K154A-K162A, plasmid pQE-CbiA K154A and for simultaneous mutations K154A-K162A-K188A, plasmid pQE-CbiA K154A-K162A was used as templates. For the generation of CbiA harboring four amino acid substitutions, plasmid pQE-CbiA K154A-K162A-K188A was applied. Double mutant K154A-R156A was randomly generated by using primers CbiA_R156A FWD and CbiA_R156A REV. Following PCR amplification and incubation with DpnI, reactions were used to transform *E. coli* cells. Plasmid DNA was isolated from selected clones and sequenced to ensure they contained the desired amino acid substitutions. All recombinant proteins were produced in *E. coli* JM109, following induction with isopropyl-β-D-thiogalactopyranoside. Cells were harvested, and lysed with a MICCRA D-9 dispersion device (Art Prozess- and Labortechnik, Müllheim, Germany) in lysis buffer containing 10 mM Imidazole, 300 mM NaCl, 50 mM NaH_2_PO_4_ and 1 mg/ml lysozyme (pH 8.0). Following centrifugation to clear cell debris, proteins were purified using Amintra Ni-NTA resin (Expedeon, Cambridge, UK). 10% Tris/Tricine SDS-PAGE followed by silver staining was used to assess purity of the samples. Protein concentrations were determined by bicinchoninic acid protein assay (Life Technologies, Darmstadt, Germany).

### SDS-PAGE, western blotting and silver staining

Recombinant proteins (500 ng each) were separated by reducing 10% Tris/Tricine SDS-PAGE and transferred to nitrocellulose membranes. Following protein transfer, membranes were blocked with 5% nonfat dry milk powder in TBS containing 0.1% Tween 20. After three wash steps with 0.1% TBS-T, membranes were probed with an anti-His antibody (1:3,000) followed by horseradish peroxidase (HRP)-conjugated anti-mouse immunoglobulins (1:1,000). Immune complexes were detected using tetramethylbenzidine (TMB) as substrate. In addition, recombinants proteins separated through a 10% Tris/Tricine SDS-PAGE were visualized by silver staining.

### Enzyme-linked immunosorbent assay (ELISA)

MaxiSorp 96-well microtiter plates (Nunc) were coated with 100 μl of recombinant proteins or BSA (5 μg/ml) in PBS at 4°C overnight with gentle agitation. Following three wash steps with PBS containing 0.05% (v/v) Tween 20 (PBS-T), wells were blocked with blocking buffer III BSA (AppliChem, Darmstadt, Germany) for 2 h at RT. Wells were washed three times with PBS-T and incubated with 100 μl plasminogen (10 μg/ml) at RT for 1 h. Following incubation, wells were washed thoroughly with PBS-T and incubated with a polyclonal goat antiserum raised against human plasminogen (1:1,000) for 1 h at RT. After washing three times with PBS-T, wells were incubated with HRP-conjugated anti-goat immunoglobulins (1:2,000) at RT for 1 h. The reaction was developed with *o*-phenylenediamine (Sigma-Aldrich, Steinheim, Germany) and the absorbance was measured at 490 nm using an ELISA reader (PowerWave HT, Bio-Tek Instruments, Winooski, VT, USA) and the Gen5 software (Bio-Tek Instruments).

The role of lysine residues in plasminogen binding was investigated by addition of increasing amounts of the lysine analog tranexamic acid (Sigma-Aldrich). The effect of increasing ionic strength on the CbiA-plasminogen interaction was determined by incubation with increasing concentrations of NaBr. To determine dose-dependency of plasminogen binding and calculate the dissociation constant, immobilized CbiA was incubated with increasing amounts of plasminogen.

### Plasminogen activation assay

Activation of CbiA bound plasminogen to plasmin was assayed using the chromogenic substrate D-Val-Leu-Lys-*p*-nitroanilide dihydrochloride. Microtiter plates (MaxiSorp, Nunc) were coated with 100 μl of recombinant proteins or BSA (5 μg/ml) in PBS at 4°C overnight. Wells were blocked with blocking buffer III BSA (AppliChem) for 2 h at RT and after washing with PBS-T, glu-plasminogen (10 μg/ml) was added. Following incubation for 1 h at RT, wells were washed three times with PBS-T and incubated with 96 μl of a reaction mixture containing 50 mM Tris/HCl, pH 7.5,300 mM NaCl, 0.003% Triton X-100, and 0.3 mg/ml S-2251. Finally, 4 μl of 2.5 μg/ml urokinase plasminogen activator (uPA) were added to activate bound plasminogen to plasmin. Microtiter plates were then incubated at 37°C and absorbance was measured every 30 min at 405 nm for a period of 24 h. In controls, either plasminogen or uPA were omitted from the reaction mixtures, or plasminogen was added together with 50 mM tranexamic acid.

### Fibrinogen degradation assay

Recombinant proteins (5 μg/ml) or BSA were immobilized in PBS on microtiter plates (MaxiSorp, Nunc) over night at 4°C. After washing with PBS-T, wells were blocked with 0.2% (w/v) BSA in PBS (PBS-BSA) for 2 h at RT. Wells were washed with PBS-T and incubated with 10 μg/ml plasminogen at RT for 1 h. Following three wash steps with PBS-T, 93.5 μl of a reaction mixture was added, containing 50 mM Tris/HCl, pH 7.5 and 20 μg/ml fibrinogen. To activate bound plasminogen to plasmin, 6.5 μl uPA (2.5 μg/ml) was added. Microtiter plates were incubated at 37°C and aliquots were taken at different time intervals (hours and minutes). Reactions were stopped by addition of SDS-PAGE sample buffer and aliquots were separated by 10% Tris/Tricine SDS-PAGE. Following transfer to nitrocellulose membranes, fibrinogen and its degradation products were visualized using a polyclonal goat anti-fibrinogen antiserum (1:1,000) and HRP-conjugated anti-goat immunoglobulins (1:1,000).

### Structural analysis

For calculation of predicted α-helices within CbiA, HcpA, BhCRASP-1, BpcA, and BtcA, the ProtScale program was used (https://web.expasy.org/protscale/) and for the calculation of the predicted coiled coils, COILS (https://embnet.vital-it.ch/software/COILS_form.html). COILS was performed with (2.5) and without weighting and windows of 14, 21, and 28.

### Statistical analysis

Unless stated otherwise, data represent means from at least three independent experiments, and error bars indicate SD. One-way ANOVA test with Bonferroni's multiple comparison post test (95% confidence interval) was employed for statistical analysis using GraphPad Prism version 7. Results were deemed statistically significant for the following *p* values: ^***^P < 0.001 and ^****^P < 0.0001.

## Results

### CbiA of *B. myiamotoi* is a plasminogen-binding protein

**Figure 1:**
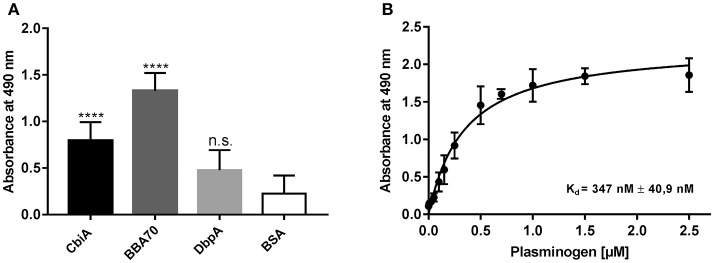
CbiA is a plasminogen-binding protein. **(A)** Binding of plasminogen to recombinant proteins as determined by ELISA. Recombinant CbiA, BBA70 (positive controls) or DbpA and BSA (negative control) (5 μg/ml each) were immobilized and incubated with 10 μg/ml plasminogen. Bound plasminogen was detected using a polyclonal antibody (1:1,000). **(B)** Dose-dependent binding of plasminogen to CbiA. Recombinant CbiA (5 μg/ml) was immobilized and incubated with increasing concentrations of plasminogen. Binding curve and dissociation constant were approximated via non-linear regression, using a one-site, specific binding model. Data represent means and standard deviation of at least three different experiments, each conducted in triplicate and compared with BSA as negative control. ****p ≤ 0.0001, n.s., no statistical significance, one-way ANOVA with post-hoc Bonferroni multiple comparison test.

CbiA has previously been identified as a novel outer surface protein of *B. myiamotoi*, exhibiting complement binding and inhibitory capacity by interacting with distinct complement components and rendering spirochetes resistant to complement-mediated lysis (Röttgerding et al., 2017). In the present study, we sought to gain insight into the molecular interaction of CbiA with plasminogen using ELISA. Initially, microtiter plates were coated with recombinant CbiA (5 μg/ml) and binding of plasminogen was detected by a specific antibody. The plasminogen-binding BBA70 protein of *B. burgdorferi* (Koenigs et al., 2013) served as a positive and DbpA of *B. burgdorferi* as a negative control protein while BSA was used as a control for unspecific binding. Both, CbiA and BBA70 bound plasminogen under non-denaturing conditions and binding to CbiA occurred in a dose-dependent manner (Figures 1A,B). Nonlinear regression allowed the approximation of the apparent dissociation constant for the CbiA-plasminogen interaction with *K*_*d*_ = 347 nM (±41 nM).

### Role of lysine residues and ionic strength in CbiA-plasminogen interaction

**Figure 2:**
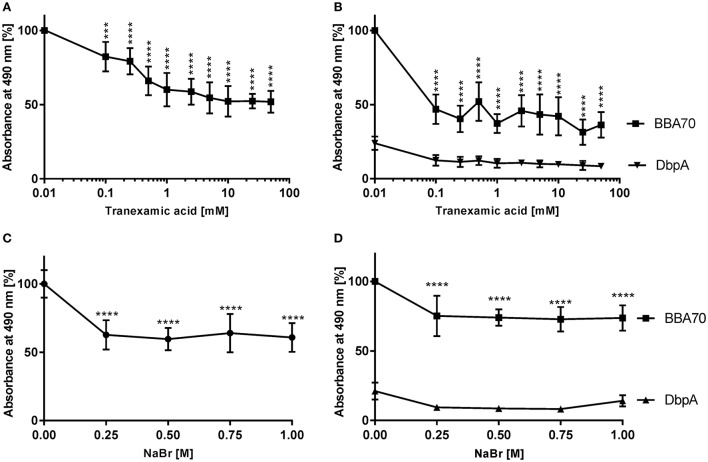
Characterization of the CbiA-plasminogen interaction. To determine the effect of the lysine analog tranexamic acid or increasing ionic strength on the CbiA-plasminogen interaction, CbiA, BBA70 (positive control), and DbpA (negative control) (5 μg/ml each) were immobilized and incubated with plasminogen in the presence of increasing concentrations of tranexamic acid **(A,B)** or NaBr **(C,D)**. Bound plasminogen was detected using a polyclonal antibody (1:1,000) and binding of plasminogen to CbiA and BBA70 in the absence of tranexamic acid or NaBr was set to 100%. Data represent means and standard deviation of at least three independent experiments, each conducted in triplicate. ****p ≤ 0.0001, one-way ANOVA with post-hoc Bonferroni multiple comparison test.

Plasminogen interacts with host proteins and receptors such as components of the ECM and fibrin or bacterial proteins via lysine-binding sites located within its kringle domains, in particular kringle domain 1, whose lysine-binding region exhibit the highest affinity among all five kringle domains (Lerch et al., 1980; Angles-Cano, 1994; Lähteenmaki et al., 2001). To investigate the role of lysine residues in the CbiA-plasminogen interaction, binding studies were conducted, using the lysine analog tranexamic acid. As shown in Figure 2, tranexamic acid significantly reduced the interaction between CbiA and plasminogen. In the presence of 0.1 mM tranexamic acid, binding of plasminogen decreased by more than 17% and decline continuously to 48% at 10 mM tranexamic acid compared to controls were the lysine analog was omitted (Figure 2A). An increase of the concentration of tranexamic acid above 10 mM had no further impact on the binding of plasminogen to CbiA. As expected, tranexamic acid strongly affected binding of BBA70 to plasminogen in the presence of 0.1 mM. In addition, the values obtained with DbpA did not change at all (Figure 2B).

Additionally, the positively charged ε-amino group of lysine residues implies that the CbiA-plasminogen interaction might be influenced through changes in ionic strength. To assess the role of electrostatic forces on binding of plasminogen to CbiA, additional binding assays were conducted in the presence of increasing concentrations of NaBr. The latter was used instead of NaCl as chloride anions promote a closed conformation of plasminogen, which might adversely affect the electrostatic interaction of both proteins irrespective of ionic strength by monovalent salts (Urano et al., 1987). At a concentration of 250 mM, a statistically significant reduction to ~ 43% in plasminogen binding could be observed (Figure 2C) suggesting that ionic strength does have an impact on the CbiA-plasminogen interaction. As previously shown, the plasminogen-BBA70 interaction was affected in the presence of increasing concentrations of NaBr (Koenigs et al., 2013; Figure 2C) while the values for DbpA remain unaffected (Figure 2D).

### Lysine and arginine residues encompassing C-terminal α-helices are not involved in the interaction with plasminogen

**Figure 3:**
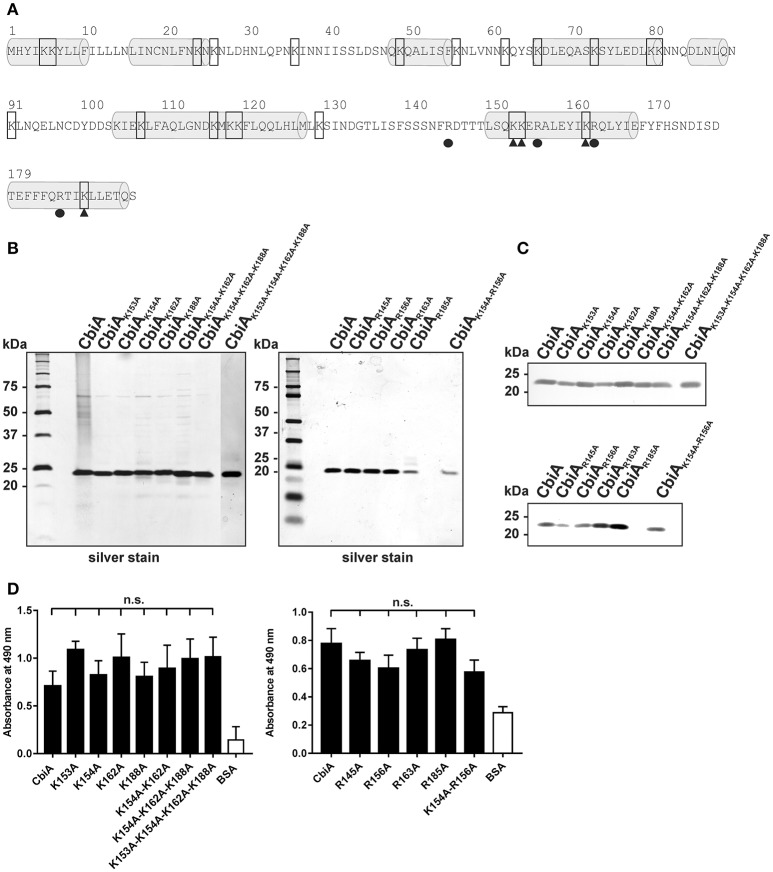
Mutational analysis of C-terminal located lysine and arginine residues of the CbiA protein. **(A)** Amino acid sequence of CbiA from *B. miyamotoi* HS1. Putative helices are highlighted by gray barrels and lysine residues are framed. The substituted lysine residues at the C-terminus are marked by filled triangles and the replaced arginine residues are indicated by filled circles. Silver staining **(B)** and Western blot analyses **(C)** were used to verify the purity of the recombinant CbiA proteins with amino acid substitutions. For Western blotting, a monospecific antibody raised against the hexahistidine-tag was used. Binding of plasminogen to CbiA proteins was determined by ELISA **(D)**. Microtiter plates were immobilized with 5 μg/ml recombinant CbiA, CbiA mutants, and BSA (negative control) and incubated with 10 μg/ml plasminogen. Bound plasminogen was detected using a polyclonal antibody (1:1,000). Data represent means and standard deviation of at least three different experiments, each conducted in triplicate. n.s., not significant. The uncropped versions of panel **(C)** is presented in Supplementary Figure 1.

To further assess the nature of the CbiA-plasminogen interaction, we sought to narrow down the plasminogen interacting region within CbiA. It has previously been shown that lysine residues located at the C-terminus of a number of bacterial proteins are involved in the interaction with plasminogen (Bergmann et al., 2001; Brissette et al., 2009; Hallström et al., 2010; Koenigs et al., 2013). Considering that lysine residues at the C-terminus often play a role in binding of plasminogen, we aimed to introduce site-directed mutations to four lysine residues encompassing position 153, 154, 162, and 188 (Figure 3A) to overcome the unforeseen technical limitations presented by generating C-terminally truncated CbiA variants (Röttgerding et al., 2017). Given that plasminogen binding is determined by intact structure, we did not intentionally target any residues with mutations likely to result in destabilization of the entire fold of CbiA. Thus, all lysine residues located within two predicted α-helices at the C-terminus were substituted by alanine to generate four single (K153A, K154A, K162A, and K188A), a double (K154A-K162A), a triple CbiA variant (K154A-K162A-K188A), and a quadruple variant (K153A-K154A-K162A-K188A). Following Ni-NTA affinity chromatography, purity of all proteins was assessed by silver staining and Western blotting (Figures 3B,C) and plasminogen binding was assayed by ELISA. As demonstrated in Figure 3D, no significant differences were observed between the single and multiple CbiA substitution variants and the wild-type protein, suggesting that lysine residues within the C-terminus may not be involved in the CbiA-plasminogen interaction. However, we cannot with reasonable certainty exclude the possibility that lysines located far distant from the C-terminus take part in binding of plasminogen.

In contrast to many bacterial plasminogen-binding proteins, the Prp protein of *Streptococcus pyogenes* binds plasminogen via arginine and histidine residues, rather than lysine residues (Sanderson-Smith et al., 2007). Later, we decided to replace additional arginine residues at the C-terminus by alanine at position 145, 156, 163, and 185. In addition, a double mutant CbiA_K154A−R156A_ accidentally generated by PCR was also included. As depicted in Figure 3D, none of the substitutions affected plasminogen binding to the CbiA variants, indicating that arginine residues did not play a role in the interaction with plasminogen.

### CbiA-bound plasminogen can be activated to plasmin

**Figure 4:**
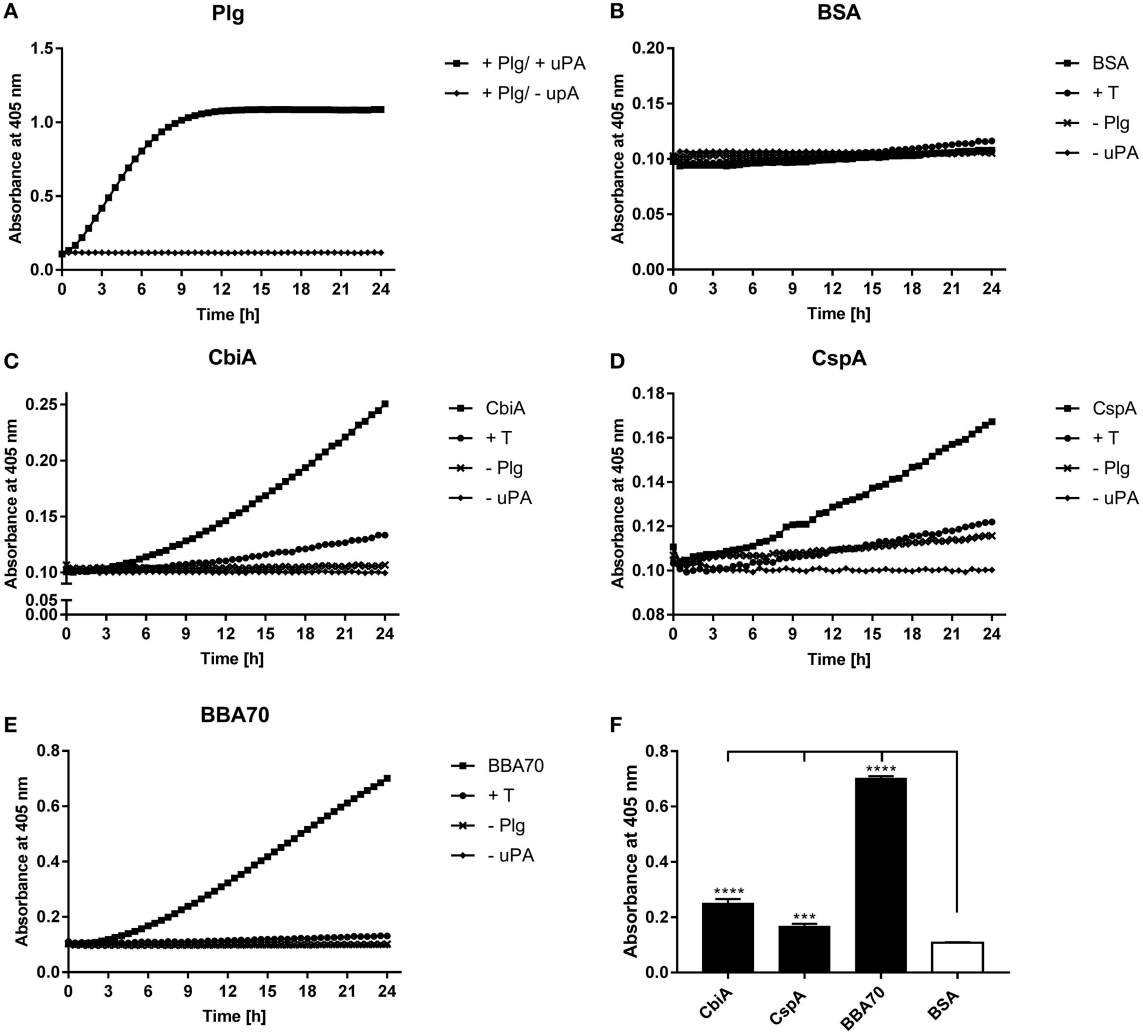
*B. miyamotoi* CbiA-bound plasminogen is converted to active plasmin by uPA. Microtiter plates were coated with 5 μg/ml of plasminogen (Plg) **(A)**, BSA **(B)**, CbiA **(C)**, CspA **(D)**, or BBA70 **(E)**. The latter four proteins were subsequently incubated with 10 μg/ml plasminogen. Following several wash steps, a reaction mixture containing the plasminogen activator uPA (final concentration of 0.1 μg/ml) and the chromogenic substrate D-Val-Leu-Lys-p-nitroanilide dihydrochloride (S-2251) was added (■). Control reactions included 50 mM of the lysine analog tranexamic acid (T) (♦) or omitted plasminogen (▾) or uPA (▴), respectively. Microtiter plates were incubated at room temperature for 24 h and absorbance at 405 nm was measured at 30 min intervals. At least three independent experiments were conducted, each in triplicate. Data shown are from a representative experiment. Evaluation of the statistical significance **(F)**. The OD values of the final measuring point (24 h) of CbiA, BBA70, DbpA, and BSA were used for the calculation using GraphPad prism 7. ***p ≤ 0.002, ****p ≤ 0.0001 compared with BSA as negative control. Raw data were analyzed by one-way ANOVA with post-hoc Bonferroni multiple comparison test.

Plasminogen can be converted to the proteolytically active serine protease plasmin by endogenous activators, such as urokinase-type plasminogen activator (uPA) or tissue-type plasminogen activator (tPA) (Dano et al., 1985) or by bacteria-secreted proteases like staphylokinase (Lijnen et al., 1994) or streptokinase (Young et al., 1998). To determine whether CbiA-bound plasminogen is accessible to uPA, microtiter plates were coated with CbiA, BBA70, CspA, or BSA, and after blocking of unspecific binding sites, incubated with plasminogen. Following washing, uPA was added together with the plasmin-specific chromogenic substrate D-Val-Leu-Lys-p-nitroanilide dihydrochloride (S-2251). As shown in Figure 4A, plasmin exhibits strong proteolytic activity to S-2251 after conversion of plasminogen in the presence of uPA, while no cleavage of the chromogenic substrate was observed when using BSA as a negative control (Figure 4B). Plasminogen bound to both CbiA as well as to BBA70 and CspA of *B. burgdorferi* used as positive controls was readily accessible to uPA and subsequently converted to active plasmin (Figures 4C–E). Additional control reactions including tranexamic acid, or omitting plasminogen or uPA, did not result in degradation of the chromogenic substrate. Degradation of S-2251 measured at the end point (24 h) was considered statistically significant (*P* ≤ 0.0001 for CbiA and BBA70, and *P* ≤ 0.001 for CspA) for the reactions containing the respective borrelial proteins, whereby plasminogen bound to BBA70 exhibited the strongest proteolytic activity (Figure 4F).

### Plasmin bound to CbiA degrades fibrinogen

**Figure 5:**
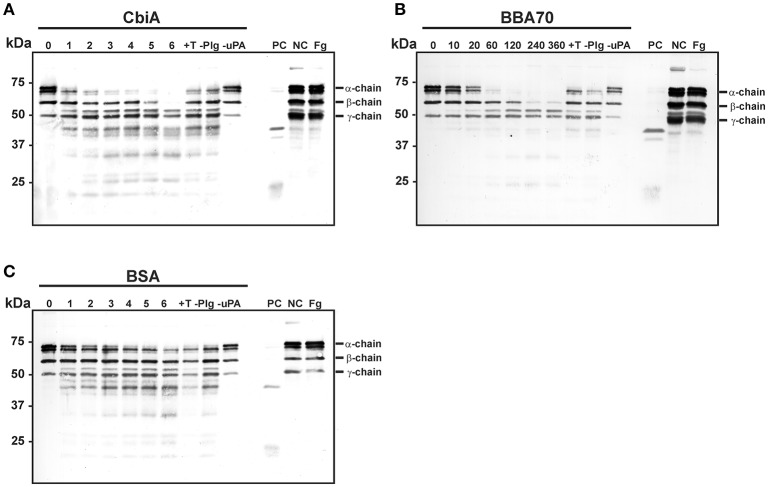
Degradation of fibrinogen by CbiA-bound plasmin. CbiA **(A)**, BBA70 **(B)**, and BSA **(C)** (5 μg/ml each) were immobilized on microtiter plates, blocked with 0.2% BSA and incubated with plasminogen (10 μg/ml). Following several wash steps, a reaction mixture containing the plasminogen activator uPA (0.16 μg/ml) and fibrinogen (20 μg/ml) was added and plates were incubated at 37°C. Samples were taken at the indicated time intervals (hour in **A,C**, and minutes in **B**) and separated via Tris/Tricine SDS-PAGE. Upon transfer to nitrocellulose membranes, fibrinogen or its degradation products were detected in a Western blot analysis using a polyclonal anti-fibrinogen antibody. Control reactions included the lysine analog tranexamic acid (+T) and omission of plasminogen (–Plg) or uPA (–uPA). Reactions containing plasminogen, uPA and fibrinogen (PC), plasminogen and fibrinogen (NC) and fibrinogen alone (Fg) were also applied as additional controls. Shown are representative results from several independent experiments. The uncropped versions of panels **(A-C)** are presented in Supplementary Figure 2.

Plasmin as a serine protease is the central component of the fibrinolytic system and degrades fibrin clots (Lijnen and Collen, 1995). We next sought to investigate, whether fibrinogen is accessible to proteolytic cleavage by CbiA-bound plasminogen. Recombinant proteins were immobilized on microtiter plates and after blocking, plasminogen was added. Following incubation, uPA as activator and fibrinogen as the physiological substrate of plasmin were added to the reactions. At several time intervals, samples were taken and degradation products were detected with a polyclonal fibrinogen antiserum employing Western blotting. The fibrinogen α-chain was completely degraded following incubation for 2–3 h and the β-chain after 6 h in reactions containing CbiA (Figure 5A). Incubation with BBA70-bound plasminogen yielded an almost complete degradation of the fibrinogen α-chain within 1 h and the β-chain within 4 h (Figure 5B). By contrast, no significant degradation was observed for BSA used as a negative control (Figure 5C). Of note, some degradation of the α-chain could be detected for control reactions at the latest time interval, including controls with the lysine analog tranexamic acid and in control reactions omitting plasminogen altogether. In summary, CbiA-bound plasminogen, upon conversion to plasmin, retained its physiological activity and was able to cleave its natural substrate fibrinogen.

## Discussion

Deciphering the biological functions of the recently identified complement inhibitory protein CbiA of *B. miyamotoi* (Röttgerding et al., 2017), here we present empirical evidence for an additional role of this surface-exposed protein as a ligand for human plasminogen. Characterizing the nature of the protein-protein interactions in more detail, we show that binding is (i) partially mediated by lysine residues, (ii) dose-dependent and (iii) affected by increasing ionic strength. More importantly, upon activation by uPA, CbiA-bound plasmin degraded its physiological substrate fibrinogen, known to be a key player in hemostasis and homeostasis, and participating in physiological extracellular matrix processes by binding to various growth factors (Bhattacharya et al., 2012). Though plasminogen may serves as a bridging molecule, CbiA by itself promotes adhesion of spirochetes to mouse brain endothelial cells (data not shown). These findings suggest that CbiA acts as a multifaceted effector molecule, enabling *B. miyamotoi* to disseminate and to overcome innate immunity, two key factors for the survival of pathogenic bacteria in an infected host.

Acquisition of plasminogen represents a common strategy developed by various human pathogenic microbes including spirochetes (Vieira and Nascimento, 2016), *Streptococcus pneumoniae* (Ullberg et al., 1990), *Neisseria meningitides* (Knaust et al., 2007), *Acinetobacter baumannii* (Lee et al., 2017), or the yeast *Candida albicans* (Marin et al., 2015) endowing pathogens with a broad-spectrum proteolytic activity. Besides characterized surface-anchored proteins, e.g., OspA, OspC, BBA70, and members of the complement regulator-acquiring surface protein family (CspA, CspZ, ErpP, ErpC, and ErpA) of *B. burgdorferi* (Fuchs et al., 1994; Brissette et al., 2009; Hallström et al., 2010; Onder et al., 2012; Koenigs et al., 2013; Hammerschmidt et al., 2014), LigA, LigB as well as Lsa23 from *L. interrogans* (Castiblanco-Valencia et al., 2016; Siqueira et al., 2016), PE protein of *Haemophilus influenza* (Barthel et al., 2012b), and Pra1 of *C. albicans* (Luo et al., 2009), proteins primarily residing in the cytoplasm may also aid in the recruitment of plasminogen, functioning as so-called moonlighting receptors on the bacterial surface. Examples include enolases from *B. burgdorferi, L. interrogans, S. pneumoniae, A. baumannii*, and *Legionella pneumophila* (Bergmann et al., 2001; Floden et al., 2011; Koenigs et al., 2015; Salazar et al., 2017). Almost all bacterial proteins whose interactions with plasminogen were biochemically analyzed exhibit a strong affinity in the nanomolar range, e.g., 23 and 125 nM for BBA70 and enolase from *B. burgdorferi*, respectively (Floden et al., 2011; Koenigs et al., 2013), 36 and 57 nM for CipA and Tuf from *A. baumannii*, respectively (Koenigs et al., 2015, 2016), and 360 nM for enolase from *Mycobacterium tuberculosis* (Rahi et al., 2017). These *K*_*D*_ values are slightly lower but comparable with that determined for the binding of plasminogen to CbiA (347 nM) (Figure 1B). Of note, the concentration of glu-plasminogen in the circulation is ~ 2 μM, thus, the interaction of CbiA with plasminogen is within the physiologically relevant range (Dano et al., 1985).

It is well known that lysine binding sites within the kringle domains of plasminogen primarily mediate binding to components of the fibrinolytic system as well as numerous bacterial proteins (Wiman et al., 1979; Lijnen et al., 1994; Bergmann et al., 2001; Koenigs et al., 2013, 2015, 2016; Hammerschmidt et al., 2014). Our findings support the notion that lysine residues play a vital role in the interaction with CbiA, as the application of the lysine analog tranexamic acid significantly reduced binding of plasminogen by up to 48% (Figure 2A) as also shown for elongation factor Tuf of *A. baumannii* (Koenigs et al., 2015). No further reduction was observed when increasing the concentration of tranexamic acid, suggesting that additional factors influence the interrelationship between plasminogen and CbiA. As expected, an increase of the NaBr concentration significantly reduced the binding of plasminogen by up to 43% indicating that the interaction is at least partially mediated by electrostatic forces (Figure 2B). Of note, NaBr was used instead of NaCl because chloride anions promote a close conformation of plasminogen and this, in turn, may render plasminogen inaccessible to bacterial ligands (Koenigs et al., 2013).

Lysine-rich motifs, often located at the C-terminus, have often been demonstrated to be an essential determinant for binding of plasminogen, for example in the case of CipA and elongation factor Tuf of *A. baumannii* (Koenigs et al., 2015, 2016), enolase of *S. pneumoniae* (Bergmann et al., 2001) or BBA70 of *B. burgdorferi* (Koenigs et al., 2013). Prediction of the secondary structure revealed that CbiA of *B. miyamotoi* HT31 consists of eight putative α-helices including the N-terminal tether peptide anchoring the molecule to the spirochetal outer membrane (Figure 3A). The primary structure of CbiA encompasses 23 (12% of the total amino acids) lysine residues. Efforts generating C-terminally truncated CbiA constructs to narrow down the plasminogen interacting site(s) failed due to the instability of the truncated CbiA proteins (Röttgerding et al., 2017). In contrast, to what would be expected from previous reports dealing with other bacterial proteins (Brissette et al., 2009; Hallström et al., 2010; Seling et al., 2010; Koenigs et al., 2016), substitutions of C-terminally located lysine residues with alanine did not influence plasminogen binding (Figure 3D). Seemingly, neither single (K153A, K154A, K162A, and K188A), nor double (K154A-K162A), triple substitutions (K154A-K162A-K188A) or quadruple substitutions (K153A-K154A-K162A-K188A) affected the interaction of CbiA with plasminogen, suggesting that the selected lysine residues are inaccessible to plasminogen or domains distal to the two C-terminal α-helices contribute to binding of the serine protease.

Bacterial plasminogen-binding proteins often contain lysine-rich motifs at the C-terminus like ErpP of *B. burgdorferi* (Brissette et al., 2009) or CipA of *A. baumannii* (Koenigs et al., 2016). Considering CbiA, the lysine residues are randomly distributed over the predicted α-helices (Figure 3A), thus, it is tempting to speculate that in the three-dimensional CbiA structure a lysine-rich motif is created after folding by bringing a constellation of otherwise distant residues into close proximity that, finally, form the plasminogen binding site.

To take into account that arginine residues might play a role in the plasminogen interaction as previously demonstrated for the Prp protein of *S. pyogenes* (Sanderson-Smith et al., 2007), we also replaced four residues at the C-terminus with alanine. All variants exhibited binding properties similar than the wild type CbiA protein, suggesting that arginine residues at these positions did not contribute to plasminogen binding.

In addition, preliminary secondary structure predictions revealed that CbiA, HcpA of *B. recurrentis*, BhCRASP-1 of *B. hermsii*, BpcA of *B. parkeri*, and BtcA of *B. turicatae* known to bind plasminogen (Rossmann et al., 2008; Grosskinsky et al., 2009; Schott et al., 2010) exhibit considerable differences in their content of α-helices and coiled coil domains, suggesting that these proteins vary in their three dimensional structures (Röttgerding et al., 2017) and Supplementary Figures 3, 4). Thus, it is tempting to speculate that structural alterations may account for the ability of these molecules to bind to plasminogen.

Of relevance, plasminogen bound to CbiA is accessible to the plasminogen activator uPA resulting in the generation of proteolytically active plasmin, which subsequently cleaved the chromogenic substrate D-Val-Leu-Lys-*p*-nitroanilide dihydrochloride (S-2251) (Figure 4) as well as the physiological substrate fibrinogen (Figure 5). The proteolytic efficiency of plasmin bound to CbiA is somewhat stronger when compared to the CspA protein of *B. burgdorferi* (Hammerschmidt et al., 2014) but four-fold lower than the BBA70 of *B. burgdorferi* known to be a very strong plasminogen-binding protein (Koenigs et al., 2013). Nevertheless, cleavage of both substrates was either completely or at nearly abrogated in control reactions lacking uPA or when tranexamic acid was added. However, some degradation of the fibrinogen α-chain was observed in the reactions omitting plasminogen altogether suggesting that trace amounts of plasminogen were present in the uPA preparation used as previously discussed (Koenigs et al., 2015). The fact that the degradation of fibrinogen was stronger in the presence of CbiA and BBA70 suggests that plasmin bound to the borrelial proteins retained its proteolytic activity, allowing for degradation of extracellular matrix components.

Lyme disease and relapsing fever spirochetes produce several plasminogen-interacting proteins, e.g., OspA, OspC, the *Borrelia*-plasminogen-binding protein BPBP, enolase, CRASPs (CspA, CspZ, ErpA, ErpC, ErpA), and BBA70 of *B. burgdorferi* (Fuchs et al., 1994; Brissette et al., 2009; Hallström et al., 2010; Floden et al., 2011; Onder et al., 2012; Koenigs et al., 2013) as well as BhCRASP-1 of *B. hermsii* (Rossmann et al., 2007), HcpA of *B. recurrentis* (Grosskinsky et al., 2009), and BpcA of *B. parkeri* (Schott et al., 2010). As multiple, surface-exposed proteins directly interact with plasminogen and thereby potentially enhance penetration of the bacterial cells through endothelial monolayers, it is, thus, not surprising that the presence of an additional plasminogen-binding protein does not cause significant differences in transmigration, in particular, if the protein is sparsely distributed on the cell surface.

Beyond its role in inhibiting complement activation by binding several complement components as well as complement regulator factor H (Röttgerding et al., 2017), binding of plasminogen by CbiA may enable *B. miyamotoi* to degrade basement membranes of the human host. Understanding the molecular mechanisms by which *B. miyamotoi* infects the human CNS will be an important step forward to learn more about the pathogenesis and tissue tropism of this newly emerging pathogen.

## Author contributions

NN: study design, data interpretation, figure preparation, and final approval. FR: study design, data interpretation, figure preparation, and final approval. GD: contribution of reagents, experimental work and final approval. Y-PL: contribution of reagents and material, critical reading of the manuscript, and final approval. AK: data interpretation, contribution of reagents and material, critical reading of the manuscript, and final approval. PK: study design, data interpretation, figure and table preparation, drafting the article, wrote the manuscript, and final approval.

### Conflict of interest statement

The authors declare that the research was conducted in the absence of any commercial or financial relationships that could be construed as a potential conflict of interest.
