# Supplementary material for: The Complement Binding and Inhibitory Protein CbiA of Borrelia miyamotoi Degrades Extracellular Matrix Components by Interacting with Plasmin(ogen)
Source: Front Cell Infect Microbiol. 2018 Feb 2;8:23. doi: 10.3389/fcimb.2018.00023 (PMC5801413; doi:10.3389/fcimb.2018.00023)
Supplement: Supplementary file 1 [file Table1.PDF]

Supplementary table 1. Oligonucleotides used in this study

Oligonucleotide	Sequence (5'-3')	Use in this work
CbiA_K153A FWD	CACAACCACTTTAAGTCAAGCAAAGGAACGAG CACTAGAATA	Generation of CbiA harboring single amino acid substitutions K153A
CbiA_K153A REV	GACGCTTTATATATTCTAGTGCTCGTTCCTTTG CTTGACTTAAAG	Generation of CbiA harboring single amino acid substitutions K153A
CbiA_K154A FWD	CACAACCACTTTAAGTCAAAAAGCGGAACGAG CACTAGAATA	Generation of CbiA harboring single amino acid substitutions K154A
CbiA_K154A REV	GACGCTTTATATATTCTAGTGCTCGTTCCGCTT TTTGACTTAAAG	Generation of CbiA harboring single amino acid substitutions K154A
CbiA_K162A FWD	CGAGCACTAGAATATATAGCGCGTCAATTATA CATTG	Generation of CbiA harboring amino acid substitutions K162A and K154A-K162A
CbiA_K162A REV	GGAAATAAAATTCAATGTATAATTGACGCGCT ATATATTC	Generation of CbiA harboring amino acid substitutions K162A and K154A-K162A
CbiA_K188A FWD	CTTCCAAAGAACAATAGCACTTTTAGAAACTC AAAG	Generation of CbiA harboring amino acid substitutions K188A and K154A-K162A-K188A
CbiA_K188A REV	CAAAAATTAACTTTGAGTTTCTAAAAGTGCTAT TGTTC	Generation of CbiA harboring amino acid substitutions K188A and K154A-K162A-K188A
CbiA_K153A/ K154A FWD	CACAACCACTTTAAGTCAAGCAGCGGAACGAG CACTAGAATA	Generation of CbiA harboring amino acid substitutions K153A- K154A-K162A-K188A
CbiA_K153A/ K154A REV	GCGCTATATATTCTAGTGCTCGTTCCGCTGCTT GACTTAAAGTG	Generation of CbiA harboring amino acid substitutions K153A- K154A-K162A-K188A
CbiA_R145A FWD	CTTTTCATCATCAAATTTTGCAGACACAACCAC TTTAAG	Generation of CbiA harboring single amino acid substitutions R145A
CbiA_R145A REV	CCTTTTTTTGACTTAAAGTGGTTGTGTCTGCAA AATTTGATG	Generation of CbiA harboring single amino acid substitutions R145A
CbiA_R156A FWD	CTTTAAGTCAAAAAAAGGAAGCAGCACTAGAA TATATAAAG	Generation of CbiA harboring single amino acid substitutions R156A
CbiA_R156A REV	GACGCTTTATATATTCTAGTGCTGCTTCCTTTTT TTGAC	Generation of CbiA harboring single amino acid substitutions R156A
CbiA_R163A FWD	CACTAGAATATATAAAGGCACAATTATACATT GAA	Generation of CbiA harboring single amino acid substitutions R163A
CbiA_R163A REV	GGAAATAAAATTCAATGTATAATTGTGCCTTT ATATATTC	Generation of CbiA harboring single amino acid substitutions R163A
CbiA_R185A FWD	CAGAATTTTTCTTCCAAGCAACAATAAAACTTT TAG	Generation of CbiA harboring single amino acid substitutions R185A
CbiA_R185A REV	GAGTTTCTAAAAGTTTTATTGTTGCTTGGAAGA AAAAT	Generation of CbiA harboring single amino acid substitutions R185A
flaB Fw_PBi	TTCAATCAGGTAACGGTACA	qRT-PCR
flaB Rv_PBi	GAAGCTTGAGATCCTGAAAG	qRT-PCR
pQE-FP-30	TTGCTTTGTGAGCGGATAAC	Sequencing
pQE-RP	CTGAGGTCATTACTGGATCTATC	Sequencing
