# Supplementary material for: The Complement Binding and Inhibitory Protein CbiA of Borrelia miyamotoi Degrades Extracellular Matrix Components by Interacting with Plasmin(ogen)
Source: Front Cell Infect Microbiol. 2018 Feb 2;8:23. doi: 10.3389/fcimb.2018.00023 (PMC5801413; doi:10.3389/fcimb.2018.00023)
Supplement: Supplementary file 2 [file Image1.PDF]

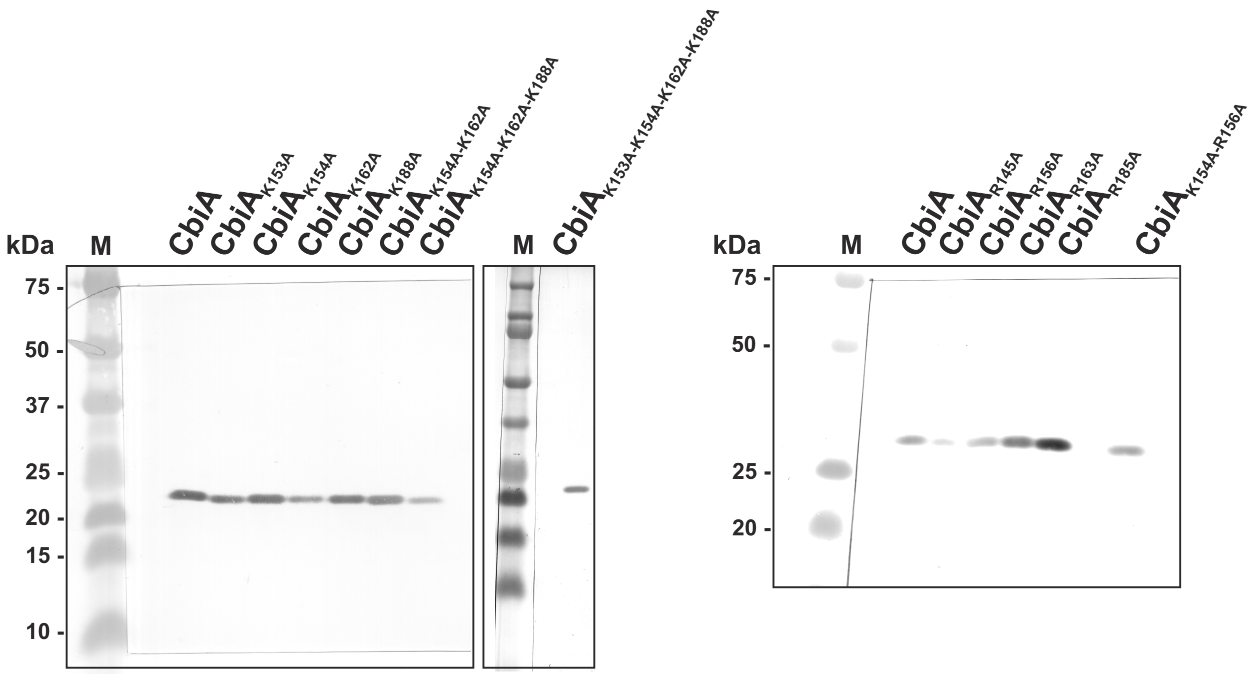

Supplementary figure 1. Identification of interacting amino acids within CbiA. Uncropped Western blot analysis of figure 3C. A monospecific antibody raised against the hexahistidine-tag was used (1:3000 dilution).
