# Supplementary material for: The Complement Binding and Inhibitory Protein CbiA of Borrelia miyamotoi Degrades Extracellular Matrix Components by Interacting with Plasmin(ogen)
Source: Front Cell Infect Microbiol. 2018 Feb 2;8:23. doi: 10.3389/fcimb.2018.00023 (PMC5801413; doi:10.3389/fcimb.2018.00023)
Supplement: Supplementary file 3 [file Image2.PDF]

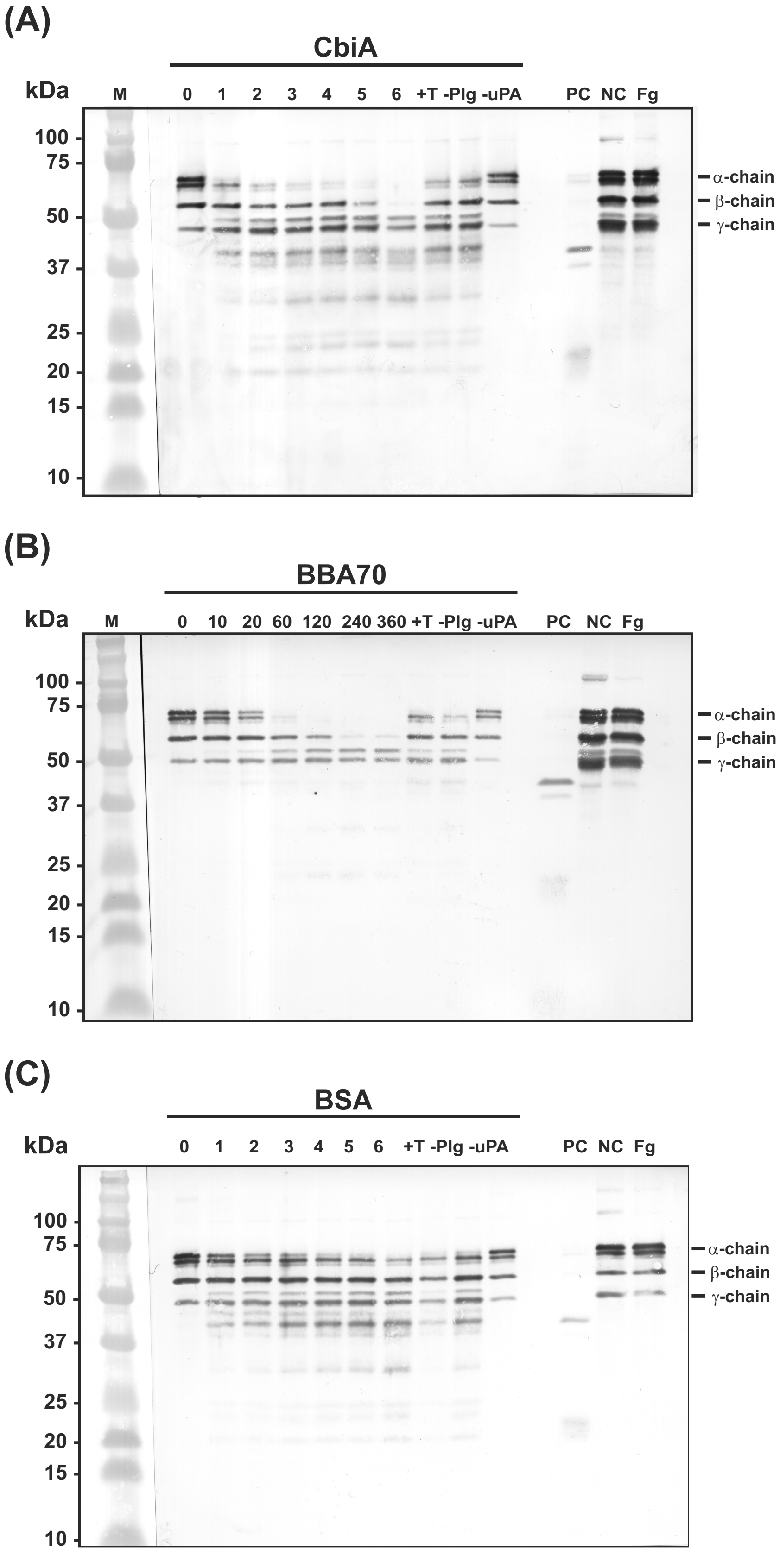

Supplementary figure S2. Degradation of fibrinogen by CbiA-bound plasmin. Uncropped Western blot analysis of figure 5. CbiA **(A)**, BBA70 **(B)** and BSA **(C)** (5 µg/ml each) were immobilized on microtiter plates, blocked with 0,2 % BSA and incubated with plasminogen (10 µg/ml). Following several wash steps, a reaction mixture containing the plasminogen activator uPA (0.16 µg/ml) and fibrinogen (20 µg/ml) was added and plates were incubated at 37 °C. Samples were taken at the indicated time intervals (hours in panel A and C, and minutes in panel B) and separated via Tris/Tricine SDS-PAGE. Upon transfer to nitrocellulose membranes, fibrinogen or its degradation products were detected in a Western blot analysis using a polyclonal anti-fibrinogen antibody. Control reactions included the lysine analog tranexamic acid (+T) and omission of plasminogen (-Plg) or uPA (-uPA). Reactions containing plasminogen, uPA and fibrinogen (PC), plasminogen and fibrinogen (NC) and fibrinogen alone (Fg) were also applied as additional controls. Shown are representative results from several independent experiments.
