# Supplementary material for: The Complement Binding and Inhibitory Protein CbiA of Borrelia miyamotoi Degrades Extracellular Matrix Components by Interacting with Plasmin(ogen)
Source: Front Cell Infect Microbiol. 2018 Feb 2;8:23. doi: 10.3389/fcimb.2018.00023 (PMC5801413; doi:10.3389/fcimb.2018.00023)
Supplement: Supplementary file 4 [file Image3.PDF]

Supplementary Figure 3

```
                                         20                    40
                                          |                     |
CbiA       M---KKYLLF ILLL-NLINC NLFNKNKNLD HNLQPNKINN 36
HcpA       M----RLYLS MALLLSIIGC ELLSKNAN-- --LQPKTLET 32
BhCRASP-1  MQLTKKYLPA ILLLLSLASC DLFYKNRNSN ANL------- 33
BpcA       MK--KKCLLT ILLL-SLVNC GLLSKNKNSE TSL------- 30
BtcA       MK--KKCLLT IILL-SLVSC GLSSKNKNSE TSLL-NIETN 36

                                         60                    80
                                          |                     |
CbiA       IISSLDSNQK QALISFKNLV NNKQYSKDLE QASKSYLEDL 76
HcpA       LESNIDDGQK QALAFFQESL QDNKHLDALK QAQSALLKDI 72
BhCRASP-1  -LKTLDNNQK QALIYFKDTL QDKKYLSYLT TSQKNFLDDL 72
BpcA       -LNTLDNNQK QALITFKDLL QDKNHRSILE KQQKSILKDL 69
BtcA       LLNTLDDNQK QALITFKDLL QDKKHLSILE KQQKSILEDL 76

                                        100                   120
                                          |                     |
CbiA       KKNNQDLNLQ NKLNQELNCD YDDSKIEKLF AQLGNDKMKK 116
HcpA       K--------- DKPQKTLNFK YNKTKLEQLF RKLGNDKTKQ 103
BhCRASP-1  EKNKKAPGLQ YKLKKTLSSE YDESQFNKLL NELGNAKAKQ 112
BpcA       EKHQENSNLQ DKLKKTLNSE YDKTQLNKLF DELGNIKTKQ 109
BtcA       KANQKNYNLQ DKLKKTLNSE YDKNQLNKLF DELGNIKTKQ 116

                                        140                   160
                                          |                     |
CbiA       FLQQLHLMLK SINDGTLISF SSSNFRDTT- TLSQKKERAL 155
HcpA       FLKNLHVTIK FINNGSFQSF SSANFNDLD- TLEKKQERAI 142
BhCRASP-1  FLQQLHIMLQ SIKDGTLTSF SSANFNDL-Q NLEQKKERAL 151
BpcA       FLQKLHIMLK SINNGTLTSF SSSNFKDSNQ TLEQKKEQAL 149
BtcA       FLQQLHIILQ SIKDGKPTNF ASSNFNNLNQ TLEQKKEQAL 156

                                        180
                                          |
CbiA       EYIKRQLYIE FYFHSNDISD TEFFFQRTIK LLETQS 191
HcpA       NSIKEELYVN YYLYTNELVD LDYFFYRAMN YL---S 175
BhCRASP-1  QSINGELYVE YYFYINGISN PDNFFEKIMQ NLKT-- 185
BpcA       QYIKGQLYTD YYLYINGIQD ANYFFERIMS VLEI-- 183
BtcA       KYIKDKLYTD YYLYINGIQD ANYFFERIMS LLE--S 190
```

Supplementary figure 3. Alignment of proteins of relapsing fever spirochetes. Deduced amino acid sequences of CbiA, BhCRASP-1, BpcA, HcpA, and BtcA were aligned using CLC Sequence Viewer, Version 8.0 (Qiagen, Aarhus). Lysine residues at the C-termini are highlighted in light grey.
