# Supplementary material for: The Complement Binding and Inhibitory Protein CbiA of Borrelia miyamotoi Degrades Extracellular Matrix Components by Interacting with Plasmin(ogen)
Source: Front Cell Infect Microbiol. 2018 Feb 2;8:23. doi: 10.3389/fcimb.2018.00023 (PMC5801413; doi:10.3389/fcimb.2018.00023)

Supplementary Figure 4

Protein	alpha helix prediction (window size: 17)	coiled coil prediction (window size: 17, 21, 28)

CbiA

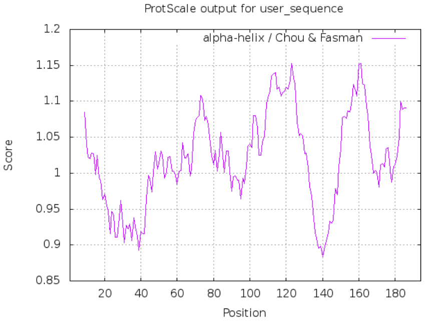

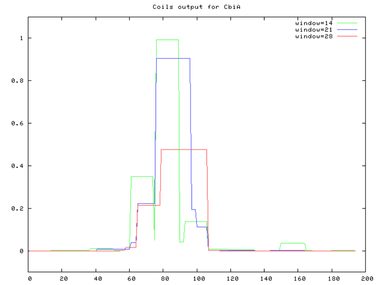

HcpA

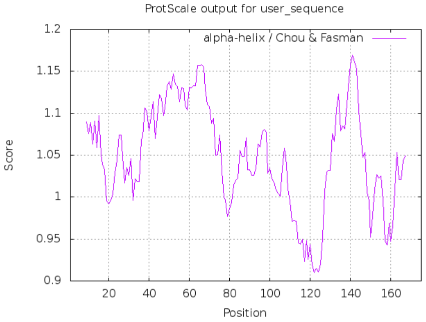

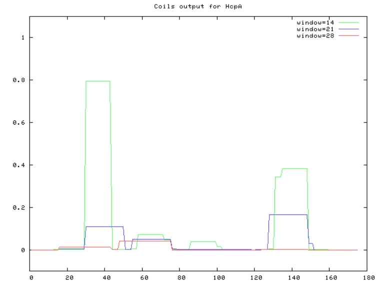

BhCRASP-1

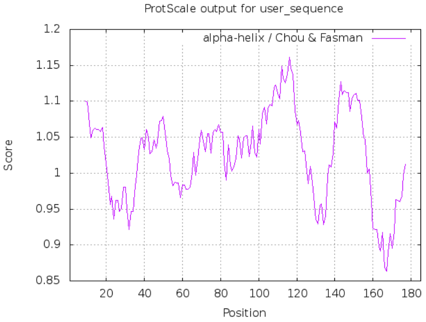

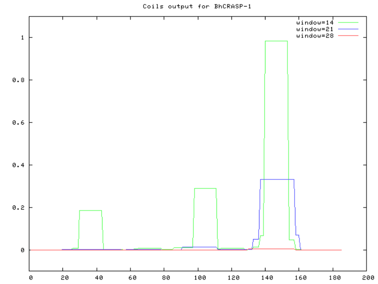

BpcA

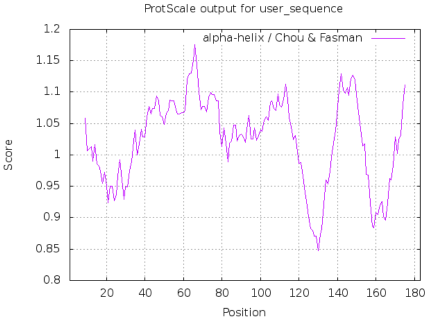

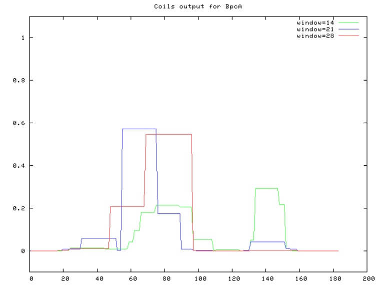

BtcA

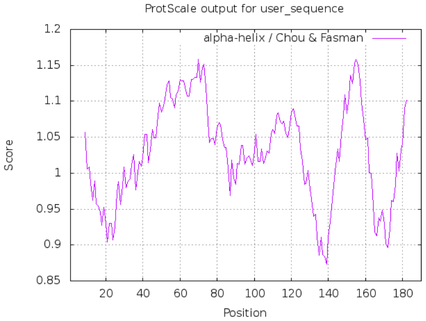

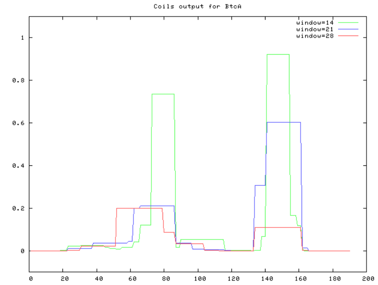

Supplement: Supplementary file 5 [file Image4.PDF]
